# Physicochemical and Functional Properties of 2S, 7S, and 11S Enriched Hemp Seed Protein Fractions

**DOI:** 10.3390/molecules27031059

**Published:** 2022-02-04

**Authors:** Comfort F. Ajibola, Rotimi E. Aluko

**Affiliations:** Department of Food and Human Nutritional Sciences, University of Manitoba, Winnipeg, MB R3T 2N2, Canada; cfajibola06@gmail.com

**Keywords:** hemp seed, globulins, albumin, amino acid composition, intrinsic fluorescence, circular dichroism, functional properties, protein digestibility

## Abstract

The hemp seed contains protein fractions that could serve as useful ingredients for food product development. However, utilization of hemp seed protein fractions in the food industry can only be successful if there is sufficient information on their levels and functional properties. Therefore, this work provides a comparative evaluation of the structural and functional properties of hemp seed protein isolate (HPI) and fractions that contain 2S, 7S, or 11S proteins. HPI and protein fractions were isolated at pH values of least solubility. Results showed that the dominant protein was 11S, with a yield of 72.70 ± 2.30%, while 7S and 2S had values of 1.29 ± 0.11% and 3.92 ± 0.15%, respectively. The 2S contained significantly (*p* < 0.05) higher contents of sulfhydryl groups at 3.69 µmol/g when compared to 7S (1.51 µmol/g), 11S (1.55 µmol/g), and HPI (1.97 µmol/g). The in vitro protein digestibility of the 2S (72.54 ± 0.52%) was significantly (*p* < 0.05) lower than those of the other isolated proteins. The intrinsic fluorescence showed that the 11S had a more rigid structure at pH 3.0, which was lost at higher pH values. We conclude that the 2S fraction has superior solubility, foaming capacity, and emulsifying activity when compared to the 7S, 11S, and HPI.

## 1. Introduction

The global demand for food proteins continues to grow and is expected to generate an estimated $76.48 billion in revenue by 2027 [1]. The reasons for increased demand for food-derived proteins have been associated with their nutritional and techno-functional properties and health benefits [2]. Thus, food proteins have become prominent ingredients in the food industry. Recently, there has been a growing interest in hemp seed proteins due to their high nutritional properties such as high digestibility and contents of sulfur-containing amino acids and arginine [3,4,5,6]. The main storage protein in hemp seed is edestin (globulins), which accounts for 60–80% of the total protein, while albumins constitute approximately 25% [7]. Currently, the available hemp seed proteins in the market are mainly defatted flours and protein concentrates, which are produced from cold-pressed seeds to remove the oil. Although hemp seed protein flours and concentrates have been successfully incorporated into a variety of products such as protein shakes, hemp milk, energy bars, and defatted meals, their use as ingredients in food applications is still limited due to poor functional properties [3,4,8].

Previous works have studied the potential use of hemp seed proteins as functional ingredients in food formulation. For example, Tang et al. [9] examined the functional properties of hemp seed protein isolate (86.9% protein content) obtained from defatted meal using alkaline solubilization followed by isoelectric precipitation at pH 5.0. With the exception of methionine and cysteine, the protein isolate contained levels of essential amino acids that satisfy human nutrition requirements. The effect of limited enzymatic hydrolysis on the functional properties of hemp seed protein was carried out by Yin et al. [10]. Malomo and Aluko [11] compared the functional properties of a protein concentrate obtained by membrane ultrafiltration (after the digestion of the defatted meal using carbohydrase and phytase to remove non-protein materials) with those of a protein isolate obtained by isoelectric precipitation. A comparative study of the structural and functional properties of hemp seed albumin and globulin protein fractions was carried out by Malomo and Aluko [12]. Wang et al. [13] studied the physicochemical and nutritional properties of hemp seed 11S (legumin), which had an isoelectric point at pH 6.4 when compared to the 7S (Vicilin) with least solubility at pH 4.6. The 7S was limited in contents of methionine and cysteine in contrast to the 11S, which contained sufficient amounts of all the essential amino acids. Dapcevic-Hadnadev et al. [3] recently reported the effect of protein isolation method on the emulsifying properties of hemp seed proteins. However, to the best of our knowledge, information is scant on the comparative structural and functional properties of a hemp seed fractions enriched in 11S, 7S, and 2S proteins. The present work presents new information on the physicochemical and functional properties of HPI, 11S, 7S, and 2S hemp seed proteins, which could promote their use as ingredients to formulate novel food products. Hence, the aim of this study was to determine the structural and functional properties of hemp seed extracts enriched with 11S, 7S, and 2S proteins in comparison to the protein isolate.

## 2. Results

### 2.1. Proximate Composition

The proximate compositions of HPI, 11S, 7S, and 2S are shown in Table 1. The moisture content was significantly higher in the 2S protein fraction, which could have contributed to the reduced fat level when compared to HPI, 11S, and 7S. The HPI and 11S had significantly higher crude protein content, which indicates greater protein purity when compared to the 2S and 7S protein fractions. Fat content was highest in the 11S followed by the 7S, while the lowest level was present in the 2S fraction. The 11S had the significantly lowest ash content, which indicates the presence of lower amounts of mineral compounds when compared to the HPI, 2S, and 7S proteins. In general, all the proteins had very low (<1.5%) fiber contents and the 2S was almost devoid of this non-nutrient polysaccharide.

### 2.2. Yield, Digestibility, Sulfhydryl Group, and Bound Carbohydrate

Table 2 shows that the 11S globulin is the predominant protein in hemp seed, accounting for approx. 73% of the total proteins while the 7S and 2S can be considered as minor proteins. In the present study, a combination of proteases was used to simulate the gastrointestinal enzymatic process that occurs in the normal human digestion of food proteins. Results show that the 2S protein had significantly lower digestibility than the HPI, 11S, and 7S proteins (Table 2). HPI and 11S had similar protein digestibility, though higher than that of 7S. However, the 2S protein had significantly higher levels of exposed and total sulfhydryl groups when compared to the HPI, 11S, and 7S. The 11S had the lowest number of exposed sulfhydryl groups as well as bound carbohydrates, but the 2S and 7S contained similar contents of bound carbohydrates, which were higher than that of the HPI.

### 2.3. Amino Acid Composition

Table 3 shows that the amino composition of the 2S protein (albumins) differs from those of 7S, 11S, and HPI (mainly globulins). The 2S had lower contents of aspartic + asparagine (Asx), phenylalanine, tyrosine, and branched-chain amino acids (valine, leucine, isoleucine) but higher contents of glutamic + glutamine (Glx) and cysteine. Except for a slightly higher level of sulfur-containing amino acids (SCAAs), the amino acid composition of the 11S was similar to that of the HPI. The 11S and HPI also had higher levels of Arg/Lys ratios when compared to the 2S and 7S protein fractions. The total level of aromatic amino acids (AAA), hydrophobic amino acids (HAA), and essential amino acids (EAA) were lower in the 2S than the 7S, 11S, and HPI. With the exception of threonine, histidine, and lysine, the 2S had essential amino acid levels that do not satisfy the minimum requirement for children. In contrast, the 11S and HPI were deficient only in lysine, while the 7S had levels of essential amino acids that meet the FAO-suggested levels for children.

### 2.4. Gel Electrophoresis (SDS-PAGE)

**Figure 1:**
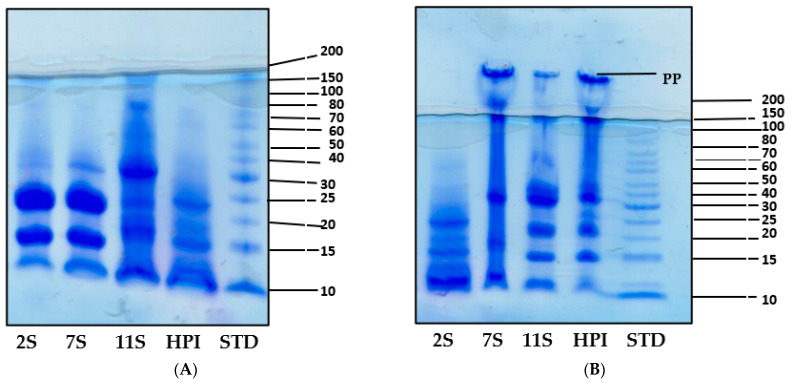
SDS-PAGE of hemp seed protein isolate (HPI) and fractions (2S, 7S, and 11S) under reducing (**A**) and non-reducing (**B**) conditions.

The SDS-PAGE profiles of the polypeptide components of HPI, 11S, 7S, and 2S in the presence (reduced) and absence (non-reduced) of mercaptoethanol are presented in Figure 1A,B, respectively. The 2S profile under non-reduced conditions had five major polypeptides that are <30 kDa while the 7S, 11S, and HPI consisted of additional polypeptides with up to 150 kDa in size. The 7S profile under non-reduced conditions confirmed the presence of four major polypeptides (150, 100, 49, and 15 kDa). The non-reduced 11S and HPI had a similar five polypeptide bands with a basic subunit (18 to 20 kDa) and an acidic subunit (30 to 40 kDa) and other polypeptides showing MW values of 47, 80, 120, and 160 kDa. The similarity of the polypeptide composition of 11S and HPI is consistent with the dominant role of 11S as the major (approx. 73%) hemp seed protein (Table 2). Moreover, the 7S, 11S, and HPI all contained polymeric proteins that could not enter the gel (PP) under non-reduced conditions (Figure 1B), which indicates protein aggregation and a hydrophobic character. In contrast, the 2S did not contain the polymeric aggregates (PP) and is an indication of a hydrophilic protein. Under the reduced condition, the 2S fraction had two major polypeptides (20 and 25 kDa) along with three minor bands, which present a different pattern when compared to the non-reducing condition and is an indication of the presence of disulfide bonds in the native protein. Similarly, the polypeptide profiles of 7S, 11S, and HPI under reduced conditions were different from those of non-reducing conditions, which also confirm the presence of disulfide bonds in the native forms of these proteins. The protein aggregates (PP) observed for 7S, 11S, and HPI under non-reduced conditions disappeared under reduced conditions, which indicate conversion into smaller monomeric polypeptides after the disruption of the disulfide bonds.

### 2.5. Intrinsic Fluorescence Emission

**Figure 2:**
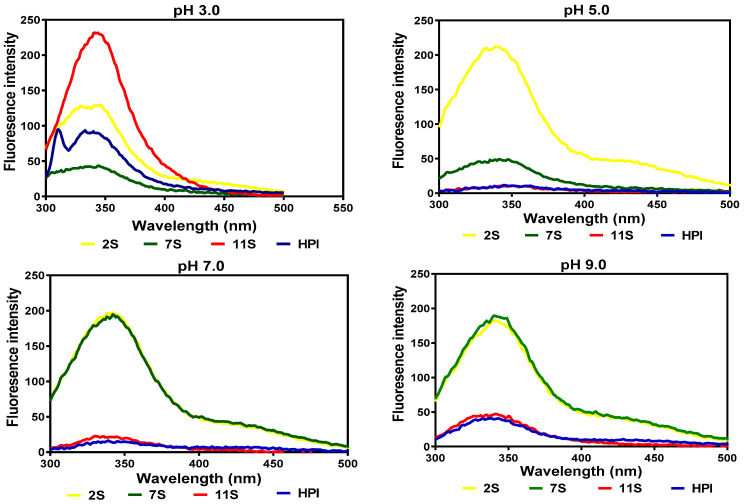
Intrinsic fluorescence intensity of hemp seed protein isolate (HPI) and fractions (2S, 7S, and 11S).

The fluorescence intensity (FI) of the hemp seed proteins was maximal (λmax) at 338–344 nm at all the pH values (Figure 2). At pH 3.0, the 11S fraction exhibited a more compact structure, which is reflected in the higher FI when compared to the 2S, 7S, and HPI. At pH 5.0 and 7.0, the 11S and HPI assumed disorganized structures, which increased interactions with the hydrophilic environment, as evident in their low FI values (indicative of fluorescence quenching), while there was a slight increase at pH 9.0. In contrast, the 2S and 7S had less compact structures (lower FI) at pH 3.0 but were significantly enhanced (higher FI) at pH 5.0, 7.0, and 9.0.

### 2.6. Secondary and Tertiary Structure Conformations

**Figure 3:**
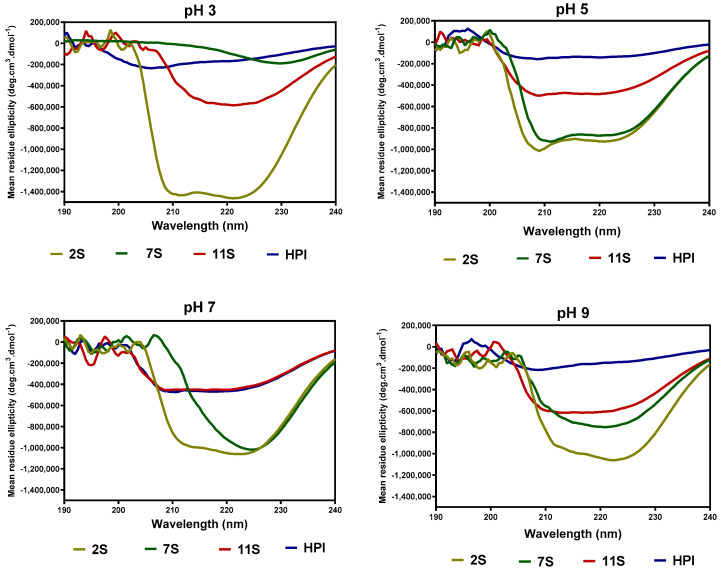
Far-UV spectra of hemp seed protein isolate (HPI) and fractions (2S, 7S, and 11S) at different pH values.

The effect of pH on the secondary structure conformations of hemp seed proteins are shown as changes in ellipticity values (Figure 3) and proportions of each structural type (Table 4). At pH 3.0, the 2S had a secondary structure dominated mostly (80%) by the α-helix conformation (Table 2), as is also evident in the intense ellipticity between 200 and 220 nm (Figure 3) when compared to 7S, 11S, and HPI. As the pH increased, the 2S lost most of the α-helix conformation accompanied by high levels of the β-sheet and unordered conformations. In contrast, the secondary structure of 7S, 11S, and HPI proteins was dominated mainly by β-sheet and unordered conformations at all the pH values (Table 2).

**Figure 4:**
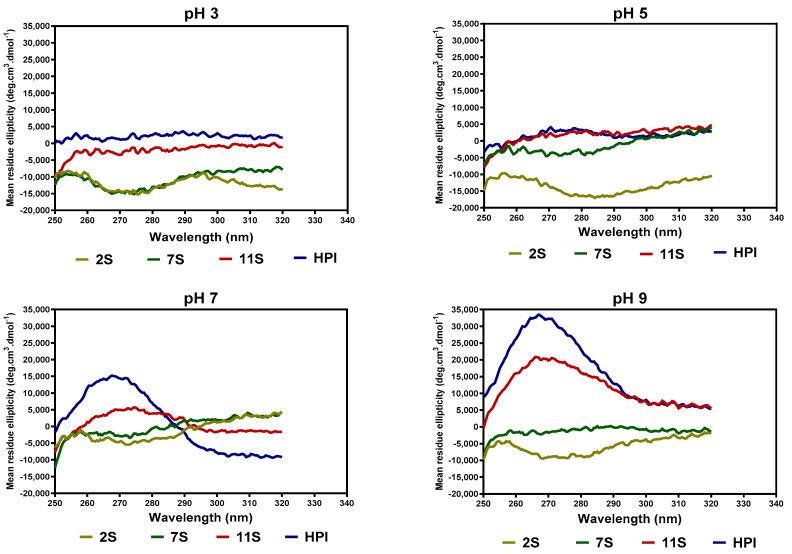
Near-UV spectra of hemp seed protein isolate (HPI) and fractions (2S, 7S, and 11S) at different pH values.

The pH-dependent tertiary structure conformations of the hemp seed proteins, as analyzed by near-UV CD spectroscopy, are shown in Figure 4. At pH 3.0, the 2S and 7S had a more organized tertiary structure (increased ellipticity between 260 and 290 nm) when compared to 11S and HPI. At pH 5.0, there was a slight increase in the compactness of the 2S protein, as evident in the increased ellipticity, whereas the 7S lost a significant part of the compact structure and hence had reduced ellipticity values when compared to pH 3.0. At pH 7.0, there was a significant increase in the ellipticity values of HPI, which reflects a more compact structure when compared to 2S, 7S, and 11S.

### 2.7. Protein Solubility Profiles

**Figure 5:**
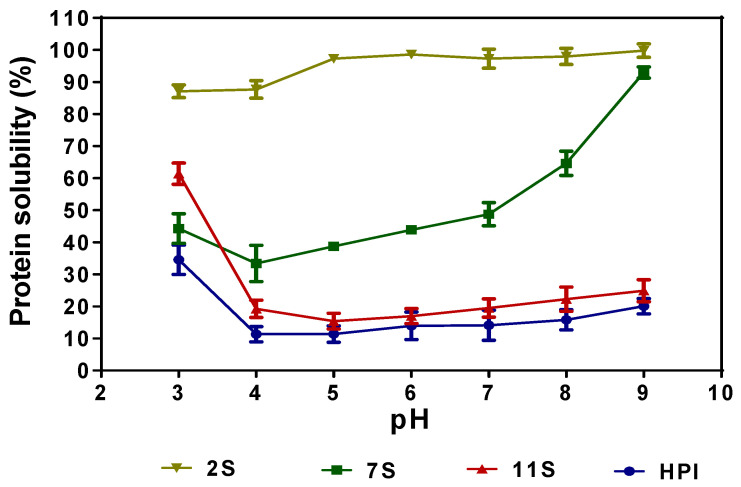
pH-dependent changes in the solubility of hemp seed protein isolate (HPI) and fractions (2S, 7S, and 11S).

Figure 5 shows the pH-dependent solubility of 11S, 7S, 2S, and HPI. The results indicate that HPI and 11S had similar solubility profiles in which the proteins have moderate (30–60%) solubility at pH 3.0, which decreased significantly (*p* < 0.05) at pH 5.0–9.0. The PS profile of 7S shows that the protein is fairly soluble at pH 3.0 and 4.0, characterized by minimal solubility around the isoelectric point, and higher solubility at pH 5.0–9.0. The 2S fraction was highly soluble over a wide pH range, with values reaching 97–99% at pH 5.0–9.0.

### 2.8. Water Holding Capacity (WHC), Oil Holding Capacity (OHC), and Least Gelation Concentration

The WHC of HPI was significantly (*p* < 0.05) higher than the values obtained for 7S and 11S (Table 5). The WHC of 2S was not reported due to complete solubility in water. The OHC is the ability of non-polar side chains of protein to interact with aliphatic chains of oil/fat and is usually expressed as the amount of fat/oil that can be absorbed per gram of protein. The 2S and HPI had similar OHCs, which were significantly higher (*p* < 0.05) than the values obtained for 7S and 11S. The LGC results show that the 7S has a higher ability to form a gel, hence a smaller amount of the protein is required when compared to 2S, 11S, and HPI (Table 5).

### 2.9. Foaming Capacity (FC) and Foam Stability (FS) of Hemp Seed Proteins

**Figure 6:**
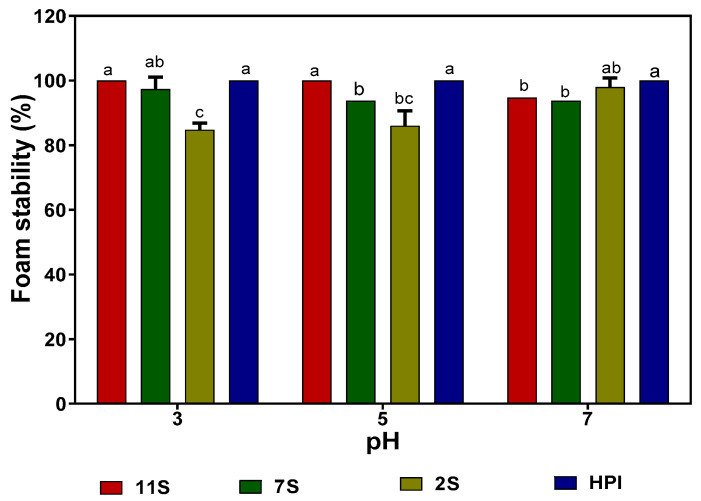
Foam stability of hemp seed protein isolate (HPI) and fractions (2S, 7S, and 11S) at different pH values. Bars with different letters have significantly different values (*p* < 0.05).

The FCs of hemp seed proteins at different pH values indicate that the 2S fraction produced significantly (*p* ˂ 0.05) larger volumes of foam than those of 7S, 11S, and HPI at all the pH values (Table 6). In general, the FCs of HPI, 11S, and 7S were minimal at pH 5.0, which is within the pH 4.5–5.0 isoelectric point range for the proteins. In contrast, the 2S had high FC in the acidic pH 3.0 and 5.0 with a slight reduction at pH 7.0. Figure 6 shows that the 2S has lower FS when compared to the 7S, 11S, and HPI, especially at pH 3.0 and 5.0. Generally, all the proteins produced stable foams (85% stability) over the 30 min measurement duration.

### 2.10. Emulsion Formation (Oil Droplet Size) and Stability

**Figure 7:**
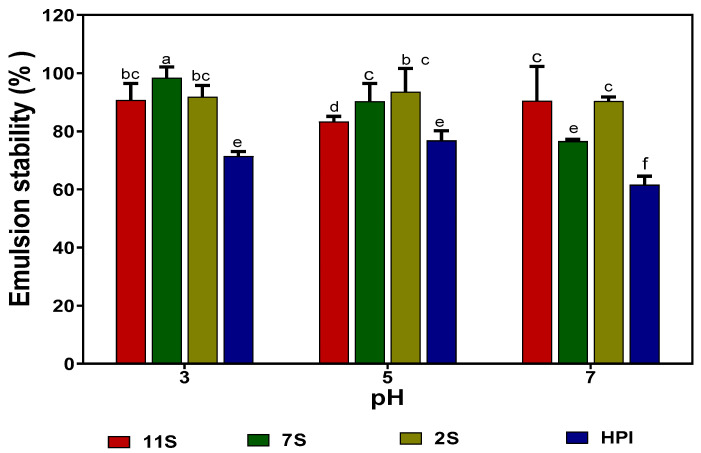
Emulsion stability of hemp seed protein isolate (HPI) and fractions (2S, 7S, and 11S) at different pH values. Bars with different letters have significantly different values (*p* < 0.05).

The emulsifying capacities of 2S, 7S, 11S, and HPI were analyzed by measuring the mean oil droplet size (*d*_3,2_) of emulsions formed by these proteins at different pH values. The 2S fraction consistently formed smaller oil droplets when compared to the 7S, 11S, and HPI proteins at all pH values (Table 7). The results show that the 2S protein has better emulsifying properties (smaller oil droplet sizes) when compared to 7S, 11S, and HPI, which formed bigger oil droplet sizes. At pH 3.0 and 5.0, the 11S had a better emulsifying capacity (smaller *d*_3,2_ values) when compared to 7S and HPI, whereas at pH 7.0, the 7S was better. The emulsions formed with 2S, 7S, and 11S proteins exhibited better stability at all pH values when compared to those formed with HPI (Figure 7).

## 3. Discussion

Knowledge of the physicochemical properties of food proteins is very important in providing a mechanistic understanding of how they may function as ingredients in food product formulations. In this work, we have provided new information on the chemical composition and structural properties of hemp seed protein fractions as a means of enhancing their utilization as food ingredients. The higher moisture content of the 2S, when compared to other fractions (Table 1), could be associated with the lower fat, which favors interactions with water molecules. The presence of attached carbohydrate molecules may have also enhanced the interactions with water molecules, as evident in the higher moisture content of 2S and 7S proteins, both of which have higher carbohydrate contents when compared to HPI and 11S (Table 2). The 87% protein content obtained for HPI is the same as the value reported by Tang et al. [9]. Wang et al. [13] had reported 90% and 93% protein contents for HPI and 11S, which are slightly higher than the 87% obtained in the present study. In contrast, the ~58% protein content of the 7S is lower than the ~88% reported by Wang et al. [13], which may be due to differences in the raw materials used for the protein extraction. The lower protein contents observed in 7S and 2S may be due to the presence of higher levels of attached carbohydrate molecules (Table 2) when compared to the 11S and HPI proteins. The results are consistent with previous works that have reported that the 2S [14,15] and 7S [16] proteins of different legume seeds are glycoproteins with covalently bound carbohydrate moieties. The 11S had the lowest ash content, which suggests the presence of lower amounts of mineral compounds when compared to the HPI, 2S, and 7S proteins. The ash contents of the HPI and 7S are higher than the <0.4% values reported by Wang et al. [13].

Table 2 shows that the 11S globulin is the predominant protein in hemp seed, accounting for approx. 73% of the total proteins while the 7S and 2S can be considered as minor proteins. This is in contrast to some legume seeds where the 7S is more abundant than the 11S [17,18]. Using a similar isoelectric protein precipitation method, Tang et al. [9] reported approx. 73% while Hadnadev et al. [4] and Shen et al. [6] obtained ~51% and ~47% protein yields, respectively, for HPI, which are lower than the ~83% obtained in the current work. The higher HPI yield obtained in this work may be due to the lab-scale defatting at room temperature, which would have produced a meal with reduced protein denaturation when compared to the 40 °C meal used by Tang et al. [9]. The effect of raw material quality is further demonstrated by the approx. 38% HPI yield reported by Malomo et al. [8], which was produced from a meal obtained by mechanical press-defatted hemp seed meal.

In the present study, in vitro protein digestibility was determined to estimate susceptibility to gastrointestinal proteases and hence amino acid bioavailability during dietary consumption. The 2S had lower digestibility, which could be associated with its high content of total sulfhydryl groups (Table 2) when compared to HPI, 11S, and 7S proteins. The 2S (albumins) contains proteins with a conserved skeleton of cysteine residues, which form several rigid intermolecular disulfide bonds that enhance stability to proteolytic attack [19]. House et al. [5] reported protein digestibility that ranged from 83.50 to 97.50% for hemp products, which are consistent with the values obtained for HPI, 11S, and 7S in this work. HPI and 11S had similar protein digestibility, though higher than that of 7S, which is consistent with the work of Wang et al. [13].

The 2S had the highest level of SCAAs (Table 3), which suggests a potentially better antioxidative effect than 7S, 11S, and HPI because of the suggested role of the sulfhydryl group in enhanced iron-reducing and hydrogen peroxide scavenging [20]. The levels of SCAAs obtained in this work are higher than the values reported by Wang et al. [13], which may be attributed to differences in the source of the defatted meal used for protein extraction. HPI and 11S had arginine/lysine ratios of 4.0 and 3.9, respectively, which are higher than those of 7S (1.7) and 2S (1.9). A high ratio has been reported to have a beneficial effect in lowering blood cholesterol and thereby contributing to overall cardiovascular health [21]. Therefore, the HPI and 11S may have better cardiovascular health benefits than the 2S and 7S proteins. The arginine/lysine ratios obtained in this work are consistent with the 1.74 and 4.37 reported for hemp seed albumin and globulin, respectively [12]. The HPI, 11S, and 7S (globulins) have higher contents of branched-chain amino acids (BCAAs) than the 2S (albumin), which has implications for human health. This is because BCAAs are part of indispensable amino acids and play remarkable metabolic and regulatory roles since about 40% of the total protein required by mammals and 35% of muscle protein essential amino acids are BCAAs [22]. BCAAs enhance protein synthesis, improve metabolic processes, improve immune functions, reduce oxidative stress, and improve gut health [23], which further emphasizes the higher nutritional value of HPI, 11S, and 7S when compared to the 2S. The total amount of essential amino acids (EAA) in HPI, 11S, and 7S are similar to the ~33% level suggested by the FAO/WHO for children’s health maintenance. In contrast, the 2S content of EAA is slightly below the minimum recommended level.

The polypeptide composition, as shown in Figure 1A,B, indicates similarities between 11S and HPI. The 49 kDa polypeptide in the present study is similar to the 47 kDa reported for the 7S of hemp protein by Wang et al. [13]. A previous work has also shown similar HPI polypeptides as obtained in this work under reducing and non-reducing conditions [4]. The lack of polymeric forms (PP) of the 2S could be due to the presence of a high number of sulfhydryl groups (Table 2), which enhances the greater formation of disulfide bonds and confers a more rigid structure to make the protein more resistant to protein–protein interactions when compared to the 7S, 11S, and HPI proteins.

The intrinsic fluorescence properties of a protein are determined by the location of its aromatic amino acid residues [24]. The 338–344 nm λmax obtained in this work is similar to the 344 and 340 nm reported for the 7S and 11S of soybean proteins [25]. The higher FI of the 11S protein at pH 3.0 indicates a more hydrophilic surface, which led to the packing of aromatic residues into the interior while the opposite was the case with the other protein fractions. However, at higher pH values, the presence of greater numbers of attached carbohydrate residues may have enhanced the surface hydrophilic properties of the 2S and 7S proteins, which influenced structural rearrangements that moved the aromatic residues into the non-polar interior, hence giving a higher FI when compared to the 11S and HPI. The intrinsic fluorescence data obtained for 11S and HPI in this study are in agreement with those reported for HPI by Malomo et al. [8], who observed the quenching of the FI of HPI at pH 5.0 and an increase in the FI at both acidic and alkaline pH values.

The far-UV data for 7S, 11S, and HPI show pH-dependent variations in secondary conformation, which are in agreement with the work of Choi and Ma [26], who reported that buckwheat globulins possess higher contents of β-sheet strands than α-helix at pH 3–11. This is important because high levels of β-sheet strands have been reported to be directly related to the ability of the protein to make strong gels [27]. The actual shape and magnitude of the near-UV spectrum of a protein in the region of 250 nm to 320 nm depend on the number of each type of aromatic amino acid residues, their mobility, and the nature of their environment, as well as their spatial disposition in the protein [28]. At pH 3.0, the more organized tertiary structure of 2S and 7S may be due to the presence of attached carbohydrates, which are not ionized and hence have fewer repulsions within the proteins. In contrast, the lack of a defined tertiary structure (almost zero ellipticity) for the 11S and HPI could indicate the presence of charged groups within the protein, hence strong protein–protein repulsions. At pH 7.0, the HPI had higher ellipticity values, which reflect increased interactions of the protein surface with the hydrophilic environment and the movement of the aromatic groups into the inner core of the protein when compared to the 2S, 7S, and 11S with looser structures. The 11S and HPI also had significant increases in a compact tertiary conformation at pH 9.0, which reflects increased protein surface interactions with the hydrophilic environment. An increase in negative charges as the pH moves towards alkaline pH would produce a more hydrophilic environment, which favors structural rearrangements that relocate aromatic residues into hydrophobic environments away from the protein surface.

The similar solubility profile of the HPI and 11S is consistent with their comparable polypeptide profiles and amino acid composition, which further confirms that the major proteins in hemp seed are the 11S globulins. The low solubility of 11S and HPI could be attributed to the high contents of hydrophobic amino acids, which enhance protein–protein interactions and result in protein aggregation and weak interactions with the water environment. The results are consistent with the detection of protein aggregates under non-reducing gel electrophoresis (Figure 1B), as well as previous reports that showed that hemp seed proteins exhibited poor solubility [8,9]. However, Hadnadev et al. [4] and Shen et al. [6] reported higher HPI solubility values than obtained in this work, which may be due to differences in the source of the defatted meal used for the protein isolate preparation. Moreover, the HPI used in this work was prepared through precipitation at pH 4.2, which may have led to greater protein aggregation (reduced solubility) than the pH 5.0 used in previous reports [4,6]. The improved solubility of 7S at alkaline pH may be associated with its smaller polypeptide sizes and the high content of attached carbohydrate residues, which could enhance net charge density. These structural features of the 7S protein will increase flexibility and interactions with the water environment when compared to the 11S and HPI proteins with bigger polypeptides and smaller numbers of attached carbohydrate residues. The high solubility of the 2S fraction over a wide pH range is consistent with previous reports for albumin fractions of other plant proteins [12,29]. The presence of a high level of attached carbohydrate residues (Table 2), the low molecular weight of polypeptides (Figure 1), and the low level of hydrophobic amino acids coupled with high levels of positively and negatively charged amino acids (Table 3) may have contributed to the high solubility of the 2S protein. It has also been shown that the exposure of the SH group could enhance protein interactions with water [15,30]. Hence, the higher content of exposed SH (Table 2) in the 2S could have contributed to the observed superior protein solubility when compared to the 7S, 11S, and HPI.

The higher WHC of HPI suggests a higher degree of protein aggregation (enhances trapping of water molecules) than the 7S and 11S proteins, as previously reported for aggregated soybean proteins [31]. Ajibola et al. [14] also obtained no value for the albumin fraction of African yam bean protein because of its complete solubility in water, a result that is similar to the 2S in the present work. This is consistent with a previous report stating that proteins with high solubility exhibit minimal WHC [32]. The OHC obtained for HPI in this study is comparable to the 13.7 g/g value reported by Malomo et al. [8] but higher than the 5.27 g/g reported by Tang et al. [9] for hemp seed protein isolate. The OHC of a protein has been reported to be a function of several parameters, such as the physical entrapment of oil, protein surface area, size, charge, and hydrophobicity [14]. The high OHC of HPI and 2S suggest potential use in the food industry for the formulation of meat substitutes, ground meat, baked goods, extenders, and soups.

A protein’s ability to form gels is traditionally measured by the LGC, which may be defined as the lowest protein concentration required to form a self-supporting gel that does not slide along the test tube walls in the inverted position [33]. The ability of the 7S and 2S to form gels at lower protein concentrations of 10 and 14% (*w*/*v*), respectively, could be associated with their higher solubility, smaller polypeptide sizes, increased structural flexibility, and a high percentage of attached carbohydrate residues [34]. In contrast, the poor gelling ability of 11S and HPI may be due to their poor solubility, which limits the ability of the proteins to unfold and form the required network. The results are consistent with a recent work, which showed that increased levels of the 7S pea protein (low 11S/7S ratio) produced gels at lower protein concentrations than the extract with a high 11S/7S ratio [18]. Therefore, the presence of 7S in the HPI could be responsible for the better gelling property when compared to the 11S. Likewise, the better gelling ability of the 2S may have been due to higher contents of sulfur groups, which have been shown to contribute to the formation of strong gels that are stabilized by disulfide bonds [27].

The most crucial requirement for foam formation during whipping is the ability of a surfactant to rapidly reduce the free energy (interfacial tension) and form a continuous and highly viscous film at foam interfaces [35]. The higher FC of the 2S fraction (albumin), when compared to the globulins (7S, 11S, and HPI), is consistent with previous reports for soy proteins [35] and African yam bean albumin [14]. The better FC observed in the 2S fraction might be due to its smaller polypeptides, flexibility, and high solubility index, when compared to the more globular and larger 7S, 11S, and HPI proteins. It has been reported that the higher the hydrophobicity of a protein fraction, the more stable the film that forms at the air/water interface [35]. Therefore, the high hydrophobic amino acid contents of 7S, 11S, and HPI (Table 3) may have improved protein–protein interactions to form strong interfacial membranes that reduced the coalescence rate of air bubbles (higher FS) better than the more hydrophilic 2S.

The results of the emulsion oil droplet size are in agreement with Tay et al. [35], who showed that the 2S fraction of soybean protein exhibited better emulsifying capacity when compared to 7S and 11S. The high emulsifying properties observed in 2S could be attributed to its smaller polypeptide sizes, which may have enabled rapid or more efficient rearrangement at the oil and water interface when compared to the larger 7S, 11S, and HPI polypeptides. The higher level of sulfhydryl groups could have also contributed through increased disulfide bonding to form strong interfacial membranes that enhanced oil droplet encapsulation. This is supported by a previous work, which showed that electrochemical modification of a soybean protein isolate led to an increased number of sulfhydryl groups and better emulsion formation ability [36]. Wang et al. [37] also reported that a soybean protein isolate with a higher level of free sulfhydryl groups formed smaller emulsions than the avocado protein. Since the 2S has a lower content of hydrophobic amino acids than 7S, 11S, and HPI, the results obtained in this work contrast with those previously reported for *Camellia oleifera* proteins, where protein hydrophobicity was a strong contributor to the emulsion-forming ability [38]. The better emulsifying capacity observed for the 7S protein at pH 7.0 indicates the increased ability of the protein to unfold and encapsulate oil droplet particles in a neutral environment. On the other hand, the 11S fraction has a higher ability at acidic pH values to unfold and encapsulate oil droplets. The results are consistent with the higher protein solubility of 11S at pH 3.0, which could have enhanced protein unfolding and encapsulation of the oil droplets when compared to HPI and 7S with lower solubility values. The better stability of emulsions formed at pH 3.0 and 5.0 with 2S and 7S proteins indicates the greater ability of the proteins to form stronger interfacial membranes that reduced oil droplet coalescence better than the 11S and HPI. The presence of higher numbers of exposed sulfhydryl groups in 2S and 7S proteins may also have enabled the formation of proteins with more disulfide bonds and, hence, stronger interfacial membrane integrity than those formed by the 11S and HPI.

## 4. Materials and Methods

### 4.1. Materials

Hemp seed hearts (dehulled) were purchased from Manitoba Harvest Fresh Hemp Foods Ltd. (Winnipeg, MB, Canada) and stored at −20 °C. Other analytical-grade chemicals and reagents were procured from Fisher Scientific (Oakville, ON, Canada) or Sigma Aldrich (Sigma Chemicals, St. Louis, MO, USA).

### 4.2. Preparation of Defatted Hemp Seed Flour (DHF)

Hemp seed flour was obtained by grinding the hemp seed hearts in a laboratory blender, which was followed by defatting using acetone extraction at 1:10 (*w*/*v*) for 1 h at room temperature [39]. The mixture was allowed to settle, after which the acetone was decanted. The defatting process was repeated once, after which the residual flour was air-dried in a fume hood at room temperature (23 °C) for 16 h. The resultant defatted meal was milled using a laboratory blender to obtain DHF, which was stored at −20 °C.

### 4.3. Preparation of Hemp Seed Protein Isolate (HPI)

HPI was produced from the DHF as described by Tang et al. [9], with slight modifications. The DHF was dispersed in deionized water (1:20, *w*/*v*), adjusted to pH 10.0 using 2 M NaOH, and mixed at 37 °C for 2 h; the mixture was then centrifuged (7000× *g*; 30 min, 4 °C). The supernatant was collected, adjusted to pH 4.2 with 2 M HCl to precipitate the proteins, and thereafter centrifuged (7000× *g*; 60 min; 4 °C). The resultant precipitate was washed with water, adjusted to pH 7.0 with 2 M NaOH, freeze-dried to obtain the HPI, and stored at −20 °C.

### 4.4. Preparation of 11S, 7S, and 2S Protein-Enriched Fractions

Fractions rich in 11S, 7S, and 2S proteins were prepared according to the protocols developed by Wang et al. [13]. DHF (100 g) was dispersed in distilled water (1:20, *w*/*v*) and adjusted to pH 10.0 with 2 M NaOH to solubilize the proteins at 37 °C for 2 h, followed by centrifugation (7000× *g*; 30 min; 4 °C) to obtain supernatant A. Then, NaHSO_3_ was added (0.98 g/L) to supernatant A, adjusted to pH 6.4 with 1 M HCl (to precipitate the 11S fraction), and kept overnight at 4 °C. The resultant dispersion was centrifuged (7000× *g*; 30 min; 4 °C) and the supernatant (B) saved while the precipitate (A) was adjusted to pH 7.0 with 2 M NaOH followed by dialysis against water for 3 days at 4 °C (3 water changes daily) using a 6–8 kDa molecular weight cut-off membrane. After dialysis, the precipitate A was freeze-dried as the 11S protein. The saved supernatant B was further adjusted to pH 4.6 with 2 M HCl (to precipitate the 7S fraction) and thereafter centrifuged (6500× *g*; 20 min; 4 °C) to obtain precipitate B and supernatant C. Precipitate B was adjusted to pH 7.0 with 2 M NaOH, and then dialyzed, as above, followed by freeze-drying to obtain the 7S protein. Supernatant C was also dialyzed, as above, and freeze-dried as the 2S protein. The dried 2S, 7S, and 11S enriched protein fractions were stored at −20 °C.

### 4.5. Proximate and Amino Acid Composition Analysis

The moisture, crude protein, and ash were analyzed using the relevant AOAC [40] methods. The fat and fiber contents were determined using AOCS methods [41]. The protein-bound carbohydrate content was determined as described by Mundi and Aluko [15], while the amino acid profiles were determined using previously described methods [42].

### 4.6. Determination of In Vitro Protein Digestibility

The in vitro digestibility of the proteins was determined according to the method of Hsu et al. [43], with slight modifications, using an enzyme system consisting of trypsin and chymotrypsin. A 10 mL aliquot of aqueous protein suspension (6.25 mg protein/mL) in double-distilled water was adjusted to pH 8.0 with 0.1 M NaOH while stirring at 37 °C. The enzyme solution (containing 1.6 mg/mL trypsin and 3.1 mg/mL chymotrypsin) was maintained in an ice bath and 1 mL of the solution was added to the protein suspension. The pH drop was recorded over a 10 min period and the protein digestibility of each protein sample was calculated as follows:

Protein digestibility (%) = 210.46 − 18.10X_f_ (1)

where X_f_ is the final pH value of each sample after a 10 min digestion.

### 4.7. Sodium Dodecyl Sulfate-Polyacrylamide Gel Electrophoresis (SDS-PAGE)

SDS-PAGE was performed according to the method of Ijarotimi et al. [42] using 8–25% gradient gel for polypeptide separation and development on a Phastsystem Separation and Development unit according to the manufacturer’s instructions (GE Health Sciences, Montréal, QC, Canada).

### 4.8. Total and Exposed Sulfhydryl Contents

Sulfhydryl and total cysteine contents were determined, as fully described [15]. The sulfhydryl concentration (total and exposed) in μmol/g of protein was calculated by using the extinction coefficient of 2-nitro-5-thiobenzoate at 412 nm (13,600 mol L^−1^ cm^−1^):

μmol SH/g protein = 73.53A × D/C(2)

where A = the absorbance at 412 nm; C = the sample concentration in mg solids/mL; D = dilution factor; and 73.53 is derived from 10^6^/(1.36 × 10^4^). The molar absorptivity is 1.36 × 10^4^ and 10^6^ is for conversions from the molar basis to the μM/mL basis and from mg solids to g solids.

### 4.9. Intrinsic Fluorescence Emission

The method described by Ijarotimi et al. [42] was used to record intrinsic fluorescence spectra on the FP-6300 spectrofluorimeter (Jasco Corp., Tokyo, Japan) at 25 °C with a 1 cm path length cuvette. Protein stock solution (10 mg/mL) was prepared in 0.1 M sodium phosphate buffer (pH 3.0, 5.0, 7.0, and 9.0), followed by centrifugation and the determination of the protein content of the supernatant. The supernatant was then diluted to 0.002% (*w*/*v*) and fluorescence spectra were recorded at an excitation wavelength of 275 nm (tyrosine and tryptophan) with emission recorded from 280 to 450 nm. The emission of the buffer was subtracted from that of the respective samples to obtain the reported fluorescence intensity (FI) spectra.

### 4.10. Measurements of Circular Dichroism (CD) Spectra

Far and near-UV CD spectra were measured as previously described by Ijarotimi et al. [42], using a J-815 spectropolarimeter (Jasco Corp., Tokyo, Japan) at 25 °C. Briefly, protein stock solution (10 mg/mL) was prepared in 0.1 M sodium phosphate buffer (pH 3.0, 5.0, 7.0, and 9.0), followed by centrifugation and the determination of the protein content of the supernatant. The stock solutions were each diluted to 2 and 4.0 mg/mL for far-UV and near-UV CD spectra measurement at 190–240 nm (0.5 mm quartz cell path length) and 250–320 nm (1 mm quartz cell path length), respectively. All the CD spectra were obtained as the average of three consecutive scans with the automatic subtraction of the respective buffer spectra. Secondary structure fractions were calculated from the far-UV data using the SELCON3 (optimized for 190–240 nm) secondary structure determination algorithm, as previously described [42].

### 4.11. Protein Solubility (PS)

PS was determined according to the method described by Ajibola et al. [14]. Briefly, 1 mg/mL protein dispersions were prepared in 0.1 M phosphate buffers, pH 3.0–9.0. The dispersions were vortexed for 2 min and then centrifuged (7000× *g*; 30 min; 25 °C). Protein contents in the supernatants were determined using the modified Lowry method [44] with bovine serum albumin as the standard. PS was expressed as a percentage ratio of supernatant protein content to the total protein content.

### 4.12. Water (WHC) and Oil (OHC) Holding Capacity

The WHC and OHC were determined using the method of Mundi and Aluko [15], with some modifications. The protein sample (3 g) was dispersed in 25 mL distilled water (or pure canola oil) in a 50 mL pre-weighed centrifuge tube. The dispersions were vortexed for 1 min, allowed to stand for 30 min, and then centrifuged (7000× *g*; 30 min; 25 °C). The supernatant was decanted, excess water (or oil) in the upper phase was drained for 15 min, and the tube containing the protein residue was weighed again to determine the amount of water or oil retained per gram of sample.

### 4.13. Least Gelation Concentration

The least gelation concentration was determined as previously described [45], by suspending the protein samples in water at different concentrations. The mixtures were vortexed, placed in a water bath at 95 °C for 1 h, cooled under tap water, and left in the refrigerator (4 °C) for 14 h. The sample concentration at which the gel did not slip when the tube was inverted was taken as the LGC.

### 4.14. Foaming Capacity (FC)

Foams were formed as previously described [45], using 60 mg/mL samples prepared in 5 mL of 0.01 M phosphate buffer pH 3.0, 5.0, 7.0, and 9.0, followed by homogenization at 20,000 rpm for 1 min using a 20 mm generator on the Polytron PT 3100 homogenizer (Kinematica AG, Lucerne, Switzerland). The foam was formed in a 50 mL graduated centrifuge tube, which enabled the determination of the foam volume (mL). The volume of foam remaining after standing for 30 min at room temperature was expressed as a percent value of the original foam volume to obtain the foam stability (FS).

### 4.15. Emulsion Formation and Oil Droplet Size Measurement

The oil-in-water emulsions were prepared and determined according to the method of Aluko et al. [45]. Briefly, protein samples at 50 mg/mL concentrations were prepared in 5 mL of 0.1 M phosphate buffer pH 7.0 followed by the addition of 1 mL of pure canola oil. The oil/water mixture was homogenized at 20,000 rpm for 1 min, using the 20 mm shaft on a Polytron PT 3100 homogenizer. The mean oil droplet size (*d*_3,2_) of the emulsions was determined in a Mastersizer 2000 (Malvern Instruments Ltd., Malvern, UK) with distilled water as a dispersant. Emulsions were kept at room temperature for 30 min without agitation and the increase in oil droplet size was used to determine the emulsion stability (ES).

### 4.16. Statistical Analysis

Duplicate or triplicate determinations were used to obtain mean values and standard deviations. Statistical analysis was performed with SAS (Statistical Analysis Software 9.1) using one-way ANOVA and significant differences (*p* < 0.05) were determined using Duncan’s multiple range test.

## 5. Conclusions

Hemp seed proteins were fractionated into the major globulins (7S and 11S) and albumins (2S) enriched fractions, followed by a comparison with the protein isolate (HPI). The 11S was the major fraction in hemp seed, accounting for almost 73% of the total proteins, which is responsible for the similarities to HPI with respect to the polypeptide and amino acid compositions, as well as solubility and in vitro protein digestibility. Gel electrophoresis showed that the 2S protein had polypeptides of small sizes, which could have favored better interactions with water, in addition to stronger foaming capacity and emulsifying activities. Overall, the 2S protein had the best potential as an efficient ingredient that can be used in the formulation of various food products, such as beverages, emulsions, and foams. However, the nutritional quality of the 2S is inferior to those of the 7S, 11S, and HPI. In contrast, the 7S had the highest nutritional quality and provides the best hemp seed protein choice as an ingredient to produce food gels.

## Figures and Tables

**Table 1:** Proximate composition of hemp seed protein isolate (HPI) and fractions (2S, 7S, 11S).

Sample	Moisture (%)	Protein (%)	Fat (%)	Ash (%)	Fibre (%)
HPI	4.11 ± 0.01 ^d^	87.14 ± 0.08 ^a^	2.14 ± 0.01 ^c^	8.63 ± 0.01 ^a^	0.11 ± 0.13 ^b^
11S	4.84 ± 0.03 ^c^	87.23 ± 0.04 ^a^	6.46 ± 0.06 ^a^	1.50 ± 0.17 ^c^	1.12 ± 0.47 ^a^
7S	5.18 ± 0.06 ^b^	57.70 ± 0.19 ^c^	5.33 ± 0.42 ^b^	8.66 ± 0.04 ^a^	1.04 ± 0.12 ^a^
2S	8.45 ± 0.03 ^a^	66.34 ± 0.01 ^b^	0.67 ± 0.09 ^d^	6.31 ± 0.06 ^b^	0.01 ± 0.01 ^c^

Each value is the mean and standard deviation of duplicate determinations. Within the same column, mean values with different letters are significantly different (*p* < 0.05).

**Table 2:** Yield, in vitro protein digestibility (IVPD), sulfhydryl groups (SH), and bound carbohydrates (CHO) of hemp seed protein isolate (HPI) and fractions (2S, 7S, 11S).

Sample	Protein Yield (%)	IVPD (%)	Exposed SH (µmol/g)	Total SH (µmol/g)	CHO (%)
HPI	82.72 ± 4.36 ^a^	88.10 ± 0.26 ^a^	1.16 ± 0.02 ^c^	1.97 ± 0.07 ^b^	5.16 ± 0.95 ^b^
11S	72.70 ± 2.30 ^b^	88.28 ± 0.17 ^a^	0.57 ± 0.04 ^d^	1.55 ± 0.22 ^c^	2.07 ± 0.09 ^c^
7S	1.29 ± 0.11 ^d^	84.48 ± 0.30 ^b^	1.32 ± 0.07 ^b^	1.51 ± 0.12 ^c^	10.36 ± 0.53 ^a^
2S	3.92 ± 0.15 ^c^	72.54 ± 0.52 ^c^	2.39 ± 0.14 ^a^	3.69 ± 0.05 ^a^	10.05 ± 0.49 ^c^

Each value is the mean and standard deviation of duplicate determinations. Within the same column, mean values with different letters are significantly different (*p* < 0.05).

**Table 3:** Percent amino acid composition of hemp seed protein isolate (HPI) and fractions (2S, 7S, and 11S).

Amino Acids	2S	7S	11S	HPI	FAO-/WHO-Suggested Requirements (2–5 Years)
Asx	7.50	9.15	11.04	11.60	
Thr	4.14	3.79	3.44	3.49	3.4
Ser	5.01	5.04	5.61	5.36	
Glx	25.63	20.95	18.44	18.13	
Pro	3.93	3.87	3.74	3.64	
Gly	5.75	4.17	4.06	4.18	
Ala	5.86	5.46	5.18	5.19	
Cys	4.88	2.24	1.56	1.22	
Val	3.07	4.87	4.74	5.32	3.5
Met	2.17	2.53	2.48	1.71	
Ile	1.99	3.70	3.81	4.36	2.8
Leu	4.02	6.34	6.61	6.90	6.6
Tyr	2.46	3.14	3.70	3.53	
Phe	1.43	3.67	4.49	4.78	
His	3.20	3.14	2.93	2.91	1.9
Lys	6.36	6.45	3.44	3.28	5.8
Arg	12.45	10.79	13.55	13.24	
Trp	0.18	0.70	1.19	1.14	1.1
HAA	30.86	38.45	40.00	40.80	
AAA	4.07	7.51	9.38	9.45	6.3
NCAA	33.13	30.1	29.04	29.73	
PCAA	22.01	20.38	19.92	19.43	
SCAA	7.05	4.77	4.04	2.93	2.5
EAA	26.56	35.19	33.13	33.89	32.8
BCAA	9.08	14.91	15.16	16.58	
Arg/Lys ratio	1.96	1.67	3.94	4.04	

Asx = aspartic acid + asparagine; Glx = glutamic acid + glutamine; AAA = aromatic amino acids; BCAA = branched-chain amino acids; HAA = hydrophobic amino acids; NCAA = negatively charged amino acids; PCAA = positively charged amino acids; SCAA = sulfur-containing amino acids; EAA = essential amino acids.

**Table 4:** Circular dichroism-derived protein secondary structure composition of hemp seed protein isolate (HPI) and fractions (2S, 7S, and 11S) at different pH values.

pH	Samples	α-Helix (%)	β-Sheet (%)	β-Turns (%)	Unordered (%)
pH 3	2S	80.40 ± 0.00	0.70 ±0.00	5.95 ± 0.01	1.53 ± 0.03
	7S	4.40 ± 0.00	31.18 ± 0.00	17.30 ± 0.00	46.66 ± 0.00
	11S	3.10 ± 0.01	37.45 ± 0.02	17.45 ± 0.01	43.65 ± 0.00
	HPI	2.20 ± 0.01	43.45 ± 0.03	19.45 ± 0.01	34.95 ± 0.00
pH 5	2S	18.60 ± 0.00	3.90 ± 0.01	12.50 ± 0.03	65.05 ± 0.04
	7S	1.40 ± 0.00	39.50 ± 0.00	20.35 ± 0.00	39.05 ± 0.00
	11S	1.55 ± 0.00	43.25 ± 0.02	21.30 ± 0.01	35.50 ± 0.00
	HPI	1.85 ± 0.00	41.50 ± 0.01	20.30 ± 0.00	36.35 ± 0.01
pH 7	2S	1.35 ± 0.01	37.55 ± 0.02	17.55 ± 0.00	47.05 ± 0.01
	7S	2.20 ± 0.00	40.80 ± 0.02	19.05 ± 0.03	38.85 ± 0.03
	11S	3.45 ± 0.00	44.25 ± 0.00	20.30 ± 0.00	32.20 ± 0.00
	HPI	17.40 ± 0.01	26.05 ± 0.01	20.20 ± 0.04	36.15 ± 0.04
pH 9	2S	4.55 ± 0.02	34.30 ± 0.00	17.30 ± 0.00	46.90 ± 0.00
	7S	3.30 ± 0.00	37.10 ± 0.00	17.05 ± 0.00	46.60 ± 0.00
	11S	0.00 ± 0.00	55.45 ± 0.02	23.60 ± 0.01	24.15 ± 0.00
	HPI	0.00 ± 0.00	45.95 ± 0.01	13.70 ± 0.00	39.99 ± 0.03

**Table 5:** Water holding capacity (WHC), oil holding capacity (OHC), and least gelation concentration (LGC) of hemp seed protein isolate (HPI) and fractions (2S, 7S, and 11S) *.

Sample	WHC (g/g)	OHC (g/g)	LGC (%)
HPI	5.81 ± 0.01 ^a^	10.32 ± 0.01 ^a^	22.00 ± 0.00 ^c^
11S	3.63 ± 0.03 ^c^	5.97 ± 0.08 ^b^	30.00 ± 0.00 ^d^
7S	4.09 ± 0.21 ^b^	4.93 ± 0.51 ^b^	10.00 ± 0.00 ^a^
2S		11.04 ± 0.01 ^a^	14.00 ± 0.00 ^b^

* For each column, values with different letters are significantly different (*p* < 0.05).

**Table 6:** Foaming capacity of hemp seed protein isolate (HPI) and fractions (2S, 7S, and 11S) *.

Sample	pH 3.0 (%)	pH 5.0 (%)	pH 7.0 (%)
HPI	75.00 ± 7.07 ^c^	55.00 ± 7.07 ^c^	75.00 ± 7.07 ^c^
11S	60.00 ± 0.00 ^d^	60.00 ± 0.00 ^b^	90.00 ± 0.00 ^b^
7S	95.00 ± 7.07 ^b^	60.00 ± 0.00 ^b^	60.00 ± 0.00 ^d^
2S	195.00 ± 7.07 ^a^	185.00 ± 7.07 ^a^	150.00 ± 0.00 ^a^

* For each column, values with different letters are significantly different (*p* < 0.05).

**Table 7:** Oil droplet sizes (*d*_3,2_) of emulsions formed with hemp seed protein isolate (HPI) and fractions (2S, 7S, and 11S) *.

Sample	pH 3.0 (µm)	pH 5.0 (µm)	pH 7.0 (µm)
HPI	12.65 ± 0.88 ^d^	6.23 ± 0.36 ^a^	4.90 ± 0.07 ^c^
11S	5.50 ± 0.13 ^b^	6.06 ± 0.43 ^a^	5.66 ± 0.27 ^d^
7S	7.62 ± 0.14 ^c^	6.79 ± 0.22 ^a^	4.45 ± 0.23 ^b^
2S	4.19 ± 0.17 ^a^	4.29 ± 0.20 ^b^	2.25 ± 0.01 ^a^

* For each column, values with different letters are significantly different (*p* < 0.05).

## Data Availability

Data are available from the corresponding author.

## Abbreviations

ANOVA	Analysis of variance
BCAA	Branched-chain amino acids
DHF	Defatted hemp seed flour
EAA	Essential amino acids
HPI	Hemp seed protein isolate
IVPD	In vitro protein digestibility
Sulfhydryl groups	SH
SCAA	Sulfur-containing amino acids
AAA	Aromatic amino acids
NCAA	Negatively charged amino acids
PCAA	Positively charged amino acids
HAA	Hydrophobic amino acids
Carbohydrates	CHO
FC	Foaming capacity
FS	Foam stability
ES	Emulsion stability
LGC	Least gelation concentration
WHC	Water holding capacity
OHC	Oil holding capacity
PS	Protein solubility
CD	Circular dichroism
FI	Fluorescence intensity
SDS-PAGE	Sodium dodecyl sulfate-polyacrylamide gel electrophoresis

## References

[1] Grand View Research (2020). Protein Ingredients Market Size, Share & Trends Analysis Report by Product (Plant Proteins, Animal/Dairy Proteins, Microbe-Based Proteins, Insect Proteins), by Application, by Region and Segment Forecasts. https://www.grandviewresearch.com/industry-analysis/protein-ingredients-market.

[2] Naghshi S., Sadeghi O., Willett W.C., Esmaillzadeh A. (2020). Dietary intake of total, animal, and plant proteins and risk of all cause, cardiovascular, and cancer mortality: Systematic review and dose-response meta-analysis of prospective cohort studies. BMJ.

[3] Dapcevic-Hadnadev T., Dizdar M., Pojić M., Krstonošić V., Zychowski L.M., Hadnadev M. (2019). Emulsifying properties of hemp proteins: Effect of isolation technique. Food Hydrocoll..

[4] Hadnadev M., Dapcevic-Hadnadev T., Lazaridou A., Moschakis T., Michaelidou A.M., Popovic S., Biliaderis C.G. (2018). Hempseed meal protein isolates prepared by different isolation techniques. Part I. physicochemical properties. Food Hydrocoll..

[5] House J.D., Neufeld J., Leson G. (2010). Evaluating the quality of protein from hemp seed (*Cannabis sativa* L.) products through the use of the protein digestibility-corrected amino acid score method. J. Agric. Food Chem..

[6] Shen P., Gao Z., Xu M., Ohm J.-B., Rao J., Chen B. (2020). The impact of hempseed dehulling on chemical composition, structure properties and aromatic profile of hemp protein isolate. Food Hydrocoll..

[7] Callaway J.C. (2004). Hempseed as a nutritional resource: An overview. Euphytica.

[8] Malomo S.A., He R., Aluko R.E. (2014). Structural and functional properties of hemp seed protein products. J. Food Sci..

[9] Tang C.-H., Ten Z., Wang X.-S., Yang X.-Q. (2006). Physicochemical and functional properties of hemp (*Cannabis sativa* L.) protein isolate. J. Agric. Food Chem..

[10] Yin S.W., Tang C.H., Cao J.S., Hu E.K., Wen Q.B., Yang X.Q. (2008). Effects of limited enzymatic hydrolysis with trypsin on the functional properties of hemp (*Cannabis sativa* L.) protein isolate. Food Chem..

[11] Malomo S.A., Aluko R.E. (2015). Conversion of a low protein hemp seed meal into a functional protein concentrate through enzymatic digestion of fibre coupled with membrane ultrafiltration. Innov. Food Sci. Emerg. Technol..

[12] Malomo S.A., Aluko R.E. (2015). A comparative study of the structural and functional properties of isolated hemp seed (*Cannabis sativa* L.) albumin and globulin fractions. Food Hydrocoll..

[13] Wang X.S., Tang C.H., Yang X.Q., Gao W.R. (2008). Characterization, amino acid composition and in vitro digestibility of hemp (*Cannabis sativa* L.) proteins. Food Chem..

[14] Ajibola C.F., Malomo S.A., Fagbemi T.N., Aluko R.E. (2016). Polypeptide composition and functional properties of African yam bean seed (*Sphenostylis stenocarpa*) albumin, globulin and protein concentrate. Food Hydrocoll..

[15] Mundi S., Aluko R.E. (2012). Physicochemical and functional properties of kidney bean albumin and globulin protein fractions. Food Res. Int..

[16] Kimura A., Fukuda T., Zhang M., Motoyama S., Maruyama N., Utsumi S. (2008). Comparison of physicochemical properties of 7S and 11S globulins from pea, fava bean, cowpea, and French bean with those of soybean. J. Agric. Food Chem..

[17] De Santis M.A., Rinaldi M., Menga V., Codianni P., Giuzio L., Fares C., Flagella Z. (2021). Influence of Organic and Conventional Farming on Grain Yield and Protein Composition of Chickpea Genotypes. Agronomy.

[18] Yang J., Zamani S., Liang L., Chen L. (2021). Extraction methods significantly impact pea protein composition, structure and gelling properties. Food Hydrocoll..

[19] Moreno F.J., Mellon F.A., Wickham M.S., Bottrill A.R., Mills E.C. (2005). Stability of the major allergen Brazil nut 2S albumin (Ber e 1) to physiologically relevant in vitro gastrointestinal digestion. FEBS J..

[20] Udenigwe C.C., Aluko R.E. (2011). Chemometric analysis of the amino acid requirements of antioxidant food protein hydrolysates. Int. J. Mol. Sci..

[21] Giroux I., Kurowska E.M., Freeman D.J., Carroll K.K. (1999). Addition of arginine but not glycine to lysine plus methionine-enriched diets modulates serum cholesterol and liver phospholipids in rabbits. J. Nutr..

[22] Tamanna N., Mahmood N. (2014). Emerging roles of branched-chain amino acid supplementation in human diseases. Int. Sch. Res. Not..

[23] Kawaguchi T., Izumi N., Charlton M.R., Sata M. (2011). Branched-chain amino acids as pharmacological nutrients in chronic liver disease. Hepatology.

[24] Schmid F.X., Creighton T.E. (1989). Spectra Methods of Characterizing Protein Conformation and Conformational Changes. Protein Structure: A Practical Approach.

[25] Clara Sze K.W., Kshirsagar H.H., Venkatachalam M., Sathe S.K. (2007). A circular dichroism and fluorescence spectrometric assessment of effects of selected chemical denaturants on soybean (*Glycine max* L.) storage proteins glycinin (11S) and β-conglycinin (7S). J. Agric. Food Chem..

[26] Choi S.M., Ma C.Y. (2007). Structural characterization of globulin from common buckwheat (*Fagopyrum esculentum* Moench) using circular dichroism and Raman spectroscopy. Food Chem..

[27] Zheng L., Wang Z., Kong Y., Ma Z., Wu C., Regenstein J.M., Teng F., Li Y. (2021). Different commercial soy protein isolates and the characteristics of Chiba tofu. Food Hydrocoll..

[28] Kelly S.M., Jess T.J., Price N.C. (2005). How to study proteins by circular dichroism. Biochim. Biophys. Acta.

[29] Gonzalez-Perez S., Vereijken J.M. (2007). Sunflower protein: Overview of their physicochemical, structural and functional properties. J. Sci. Food Agric..

[30] Yildiz G., Ding J., Andrade J., Engeseth N.J., Feng H. (2018). Effect of plant protein-polysaccharide complexes produced by mano-thermo-sonication and pH-shifting on the structure and stability of oil-in-water emulsions. Innov. Food Sci. Emerg. Technol..

[31] Wang X., He Z., Zeng M., Qin F., Adhikari B., Chen J. (2017). Effects of the size and content of protein aggregates on the rheological and structural properties of soy protein isolate emulsion gels induced by CaSO_4_. Food Chem..

[32] Deng Q., Wang L., Wei F., Xie B., Huang F., Huang W., Shi J., Huang Q., Tian B., Xue S. (2011). Functional properties of protein isolates, globulin and albumin extracted from *Ginkgo biloba* seeds. Food Chem..

[33] Moure A., Sineiro J., Domínguez H., Parajó J.C. (2006). Functionality of oilseed protein products: A review. Food Res. Int..

[34] Ragab D.D.M., Babiker E.E., Eltinay A.H. (2004). Fractionation, solubility and functional properties of cowpea (*Vigna unguiculata*) proteins as affected by pH and/or salt concentration. Food Chem..

[35] Tay S.L., Kasapis S., Perera C.O., Barlow P.J. (2006). Functional and structural properties of 2S soy protein in relation to other molecular protein fractions. J. Agric. Food Chem..

[36] Yu D., Zhao Y., Li T., Li D., Chen S., Wu N., Jiang L., Wang L. (2018). Effect of electrochemical modification on the structural characteristics and emulsion storage stability of soy protein isolate. Process Biochem..

[37] Wang J.-S., Wang A.-B., Zang X.-P., Tan L., Xu B.-Y., Chen H.-H., Jin Z.-Q., Ma W.-H. (2019). Physicochemical, functional and emulsion properties of edible protein from avocado (*Persea americana* Mill.) oil processing by-products. Food Chem..

[38] Yang J.-Y., Peng B., Wang M., Zou X.-G., Yin Y.-L., Deng Z.-Y. (2019). Characteristics and emulsifying properties of two protein fractions derived from the emulsion formed during aqueous extraction of Camellia oil. Food Hydrocoll..

[39] Osemwota E.C., Alashi A.M., Aluko R.E. (2021). Comparative study of the structural and functional properties of membrane-isolated and isoelectric pH precipitated green lentil seed protein isolates. Membranes.

[40] AOAC (1990). Official Methods of Analysis.

[41] AOCS (2006). American Oil Chemists’ Society Official Methods.

[42] Ijarotimi S.O., Malomo S.A., Fagbemi T.N., Osundahunsi O.F., Aluko R.E. (2018). Structural and functional properties of *Buchholzia coriacea* seed flour and protein concentrate at different pH and protein concentrations. Food Hydrocoll..

[43] Hsu H., Vavak D., Satterlee L. (1977). A multienzyme technique for estimating protein digestibility. J. Food Sci..

[44] Markwell M.A.K., Haas S.M., Bieber L.L., Tolbert N.E. (1978). A modification of the Lowry procedure to simplify protein determination in membrane and lipoprotein samples. Anal. Biochem..

[45] Aluko R.E., Mofolasayo O.A., Watts B.M. (2009). Emulsifying and foaming properties of commercial yellow pea (*Pisum sativum* L.) seed flours. J. Agric. Food Chem..

